# Effects of carnitine on oxidative stress response to intravenous iron administration to patients with CKD: impact of haptoglobin phenotype

**DOI:** 10.1186/s12882-015-0119-0

**Published:** 2015-08-13

**Authors:** Zaher Armaly, Amir Abd El Qader, Adel Jabbour, Kamal Hassan, Rawi Ramadan, Abdalla Bowirrat, Bishara Bisharat

**Affiliations:** Department of Nephrology, Nazareth Hospital-EMMS, Nazareth, 16100 Israel; Department of Laboratory Medicine, Nazareth Hospital-EMMS, Nazareth, Israel; Galilee Faculty of Medicine - Bar Ilan University, Zafed, Israel; Department of Nephrology, Western Galilee Hospital, Nahariya, Israel; Department of Nephrology, Rambam Health Campus, Haifa, Israel

**Keywords:** Anemia, Chronic kidney disease, Intravenous iron administration, Oxidative stress, Carnitine, Haptoglobin

## Abstract

**Background:**

Anemia is a common disorder in CKD patients. It is largely attributed to decreased erythropoietin (EPO) production and iron deficiency. Therefore, besides EPO, therapy includes iron replenishment. However, the latter induces oxidative stress. Haptoglobin (Hp) protein is the main line of defense against the oxidative effects of Hemoglobin/Iron. There are 3 genotypes: 1–1, 2–1 and 2–2. Hp 2–2 protein is inferior to Hp 1–1 as antioxidant. So far, there is no evidence whether haptoglobin phenotype affects iron-induced oxidative stress in CKD patients. Therefore, the present study examines the influence of carnitine treatment on the intravenous iron administration (IVIR)-induced oxidative stress in CKD patients, and whether Hp phenotype affects this response.

**Methods:**

Trial registration: Current Controlled Trials ISRCTN5700858. This study included 26 anemic (Hb = 10.23 ± 0.28) CKD patients (stages 3–4) that were given a weekly IVIR (Sodium ferric gluconate, [125 mg/100 ml] for 8 weeks, and during weeks 5–8 also received Carnitine (20 mg/kg, IV) prior to IVIR. Weekly blood samples were drawn before and after each IVIR for Hp phenotype, C-reactive protein (CRP), advanced oxidative protein products (AOPP), neutrophil gelatinase-associated lipocalin (NGAL), besides complete blood count and biochemical analyses.

**Results:**

Eight percent of CKD patients were Hp1-1, 19 % Hp2-1, and 73 % Hp2-2. IVIR for 4 weeks did not increase hemoglobin levels, yet worsened the oxidative burden as was evident by elevated plasma levels of AOPP. The highest increase in AOPP was observed in Hp2-2 patients. Simultaneous administration of Carnitine with IVIR abolished the IVIR-induced oxidative stress as evident by preventing the elevations in AOPP and NGAL, preferentially in patients with Hp2-2 phenotype.

**Conclusions:**

This study demonstrates that Hp2-2 is a significant risk factor for IVIR-induced oxidative stress in CKD patients. Our finding, that co-administration of Carnitine with IVIR preferentially attenuates the adverse consequences of IVIR, suggests a role for Carnitine therapy in these patients.

## Background

Chronic kidney disease (CKD) is a global public health problem, affecting tens of millions around the world [1, 2]. The alarming increase in the prevalence of CKD is largely attributed to the dramatic global increase of diabetes and hypertension incidence [2]. One of the major complications of CKD, especially the advanced stages of the disease, is anemia [3]. The latter is associated with poor clinical outcomes as evident by enhanced incidence of cardiovascular events and the risk of death in patients with CKD [4–6]. Thus, correction of anemia is considered as a major component in the integral therapy of CKD adverse consequences [7, 8]. The mechanisms underlying anemia in CKD patients is attributed to several factors including decreased erythropoietin (EPO) production, low iron stores, and chronic inflammation [7]. Therefore, therapy includes not only recombinant EPO, but also iron replenishment [8–10]. Intravenous iron administration (IVIR) in these CKD patients is the most efficacious and favored route of supplementation to enhance red blood cell production by recombinant human erythropoietin administration [7, 8]. There are several iron preparations for IV administration: iron sucrose, sodium ferric gluconate, low- and high molecular weight iron dextran, ferric carboxymaltose, and ferumoxytol [9, 10]. However, despite their efficacy as expressed by improved erythropoietic response in CKD subjects, exposure to IV iron is associated with enhancement of free radical generation and oxidative stress, thus aggravating the already existed oxidative burden in these patients [11–14]. There are several markers of free radical activities such as carbonyl reactive derivatives (CRD), thiol groups (SH), malondialdehyde (MDA), and antioxidant enzyme activities (superoxide dismutase, SOD and glutathione peroxidase, GPX) [15, 16]. Additional biomarker that represents plasma protein oxidation is advanced protein oxidation products (AOPP), where it has recently been applied to assess the oxidative stress and inflammation in several pathological conditions [17–20]. Enhanced oxidative stress and inflammation are known to induce direct cellular damage and possibly increasing the risk of atherosclerotic disease in CKD and hemodialysis patients [21–23]. Several studies have shown that the severity of oxidative stress-induced cardiovascular complications in various disease states is affected by haptoglobin (Hp) genotype [24, 25]. There are two classes of functional alleles Hp 1–1, 1–2 and 2–2. Hp binds to free hemoglobin (Hb) released from blood cells as part of red cell turnover, thus inhibiting the oxidative tissue damage resulting from free Hb through heme iron [26, 27]. Hp alleles differ in their ability to clear free Hb from the plasma where Hp [1]-Hb complexes are cleared more efficiently from the plasma than Hp [2]-Hb complexes [27], thus subjects with Hp 2–2 are more prone to oxidative stress [28].

Current criteria for diagnosis and classification of AKI are highly dependent on changes in SCr [29, 30]. However, in the setting of acute kidney injury (AKI), the time relationship between changes in SCr and concomitant changes in GFR do not allow accurately estimating timing of injury and severity of dysfunction [31]. Recent studies have attempted to identify by a genome-wide interrogation strategy genes that are induced very early after acute ischemia of kidney in animal models, whose protein products might serve as novel biomarkers for the initiation phase of AKI. Actually, many potential markers have been studied, including neutrophil gelatinase-associated lipocalin (NGAL), kidney injury molecule- 1 (KIM-1), and others [32, 33]. Among them, NGAL is one of the most strikingly up-regulated genes and overexpressed proteins following AKI [32]. NGAL increased in urine early after renal ischemia in mouse and rat models [34, 35], yet has not been documented in human. The additional and earlier finding that the serum and urine NGAL levels were strikingly elevated in children with AKI after cardiopulmonary bypass [35] suggests that NGAL might be a specific, and highly predictive early biomarker for AKI also in humans. Kidney injury molecule-1 (KIM-1) is a marker for proximal tubular injury, the hallmark of virtually all proteinuric, toxic and ischemic renal diseases [33, 36, 37].

Several interventions have been attempted to counteract the deleterious effects of IV induced oxidative stress in dialysis patients including administration of substances with antioxidant properties, such as $\alpha$-tocopherol, angiotensin converting enzyme inhibitors, vitamin E, N-acetyl cysteine (NAC) prior to IV iron administration [15, 38]. Unfortunately, none of these therapeutic maneuvers exerted beneficial effects against oxygen free radical injury. However, there is no doubt that any potential therapies should encounter the altered mitochondrial function. One of these approaches is the use of carnitine since it may preserve mitochondrial function and subsequently reduced oxidative burden. In line with this concept, several randomized, controlled studies and a meta-analysis have shown that L-carnitine supplementation might have a positive effect on response to EPO in long term HD patients [19, 39–42]. Unfortunately, there is no evidence whether this approach is also beneficial in earlier-stage CKD patients. Thus the present study was designed to investigate the effect of L-Carnitine therapy on oxidative stress and the inflammatory response to intravenous iron administration (IVIR) in CKD patients. In addition, we examined whether the extent of oxidative stress in these patients is affected by the Hp phenotype.

## Methods

This was a prospective, non-randomized, open-label, crossover study of the effects of IV Carnitine supplementation on oxidative stress in CKD patients receiving IV iron therapy. The study was approved by the Nazareth Hospital EMMS Human Research Review Committee and carried at Nazareth Hospital. All patients provided informed consent. Trial registration: Current Controlled Trials ISRCTN5700858. (https://clinicaltrials.gov/ct2/show/NCT02312414), Date of registration is December 5th, 2014.

### Inclusion criteria

1. Patients that have been diagnosed as suffering from chronic kidney diseases at stages 3–4 and confirmed by MDRD.
2. CKD patients with Hb of ≤ 10.2 g %.
3. At age ≥18 y.

### Exclusion criteria

1. Pregnant women.
2. Patient with CKD stage 5 on Dialysis.
3. Patients with severe liver diseases.
4. Patients with severe CHF.
5. Inter-current illness such as fever.
6. Allergic rhinitis.

### Study design

**Fig. 1:**
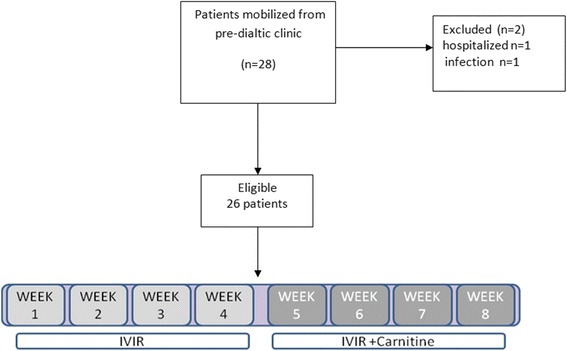
Selection of anemic CKD patients for examination of the effects of carnitine on oxidative stress response to intravenous iron administration and the impact of haptoglobin phenotype

Among 28 eligible patients 2 patients were dropped from the study due to infection and hospitalization (Fig. 1).

Sample size was determined based on the standard deviation of the obtained preliminary data. For this purpose we applied the following

$$ \begin{array}{l}{n}_1=\frac{{\left({Z}_{1-\alpha /2}+{Z}_{1-\beta}\right)}^2{\sigma}^2\left(k+1\right)}{k{\varDelta}^2}\\ {}{n}_2=k{n}_1\end{array} $$

Significance α 5 %

Confidence 1- α 95 %

SD σ 3.0

Strength β 99 %

Effect size delta 0.29

K = 1

Sample size 25

The demographic and laboratory data of the studied patients are listed in Table 1. This study included 26 anemic (Hb = 10.23 ± 0.25) CKD patients (stages 3–4) that were given by Dr. ZA at Nazareth Hospital a weekly IVIR (Sodium ferric gluconate, [125 mg/100 ml] for 8 weeks, and during weeks 5–8 also received Carnitine (20 mg/kg, IV) prior to IVIR administration). To minimize potential bias each patient served as its own control. Weekly blood samples were drawn before and after each IVIR for C-Reactive protein (CRP), advanced oxidative protein products (AOPP), neutrophil gelatinase-associated lipocalin (NGAL), in addition to routine complete blood count and biochemical analyses.

**Table 1:** Baseline Demographic and Laboratory Data of the 26 Studied Patients

Parameter	Value
Age, mean ± SD (yrs)	63.84 ± 2.84
Gender (Male : Female)	13 : 13
Etiology of CKD	
Disease (no.)	
Diabetes mellitus	18
Hypertension	16
Other	3
MDRD-GFR, (ml/min/1.73 m^2^)	33.2 ± 4.02
Stage of CKD (no.)	
Stage 3	11
Stage 4	15
Hemoglobin (g %)	10.3 ± 0.25

### Chemical and hematological analysis

#### AOPP (Advanced oxidation protein products)

A novel spectrophotometric assay which allows detecting advanced oxidation protein products (AOPP) in uremic plasma was used. AOPP levels were measured with the Witko-Sarsat method [19] of optical density analysis in patient blood samples. AOPP maximum absorption is at a wavelength of 340 nm under acidic conditions, therefor this wavelength provides the best indication of the level of the AOPP in the samples. By size-exclusion chromatography, AOPP are retrieved in two distinct peaks at 600 and below 80 kDa in uremic plasma, while no such peaks are found in control plasma.

#### Determination of NGAL

This biomarker was determined in specimens of plasma that were stored at −80 °C until analysis. Blood specimens were collected aseptically into EDTA-containing tubes, centrifuged at 3000 rpm and serum separated and stored at −80 °C until testing. Plasma level of NGAL was measured with a commercially available ELISA kit purchased from Bio Porto Diagnostics (Gentofte, Denmark).

#### Haptoglobin phenotype

Haptoglobin phenotype was determined as described by Hochberg et al. [43]. Briefly, serum (10 μL) was mixed with 2 μL of a 10 % hemoglobin solution, and the samples were incubated for 5 min at room temperature to permit the haptoglobin-hemoglobin complexes to form. The haptoglobin-hemoglobin complex was resolved by polyacrylamide electrophoresis. The haptoglobin-hemoglobin complexes were visualized by soaking the gel in freshly prepared staining solution. The bands corresponding to the haptoglobin-hemoglobin complex were readily visible within 15 min. All gels were documented with photographs. Phenotypes Hp 1–1, Hp 2–2, and Hp 2–1 were distinguished by a characteristic pattern of bands representing the haptoglobin-hemoglobin complexes.

### Statistical analysis

Data are expressed as means ± SEM. Microsoft Excel software was used to analyze data, create standard curves and draw figures. Statistical significance was assessed by one-way analysis of variance (ANOVA) for repeated measures. Tukey’s multiple comparisons test was used for data point comparisons in each group. P ≤ 0.05 was considered statistically significant.

## Results and discussion

**Fig. 2:**
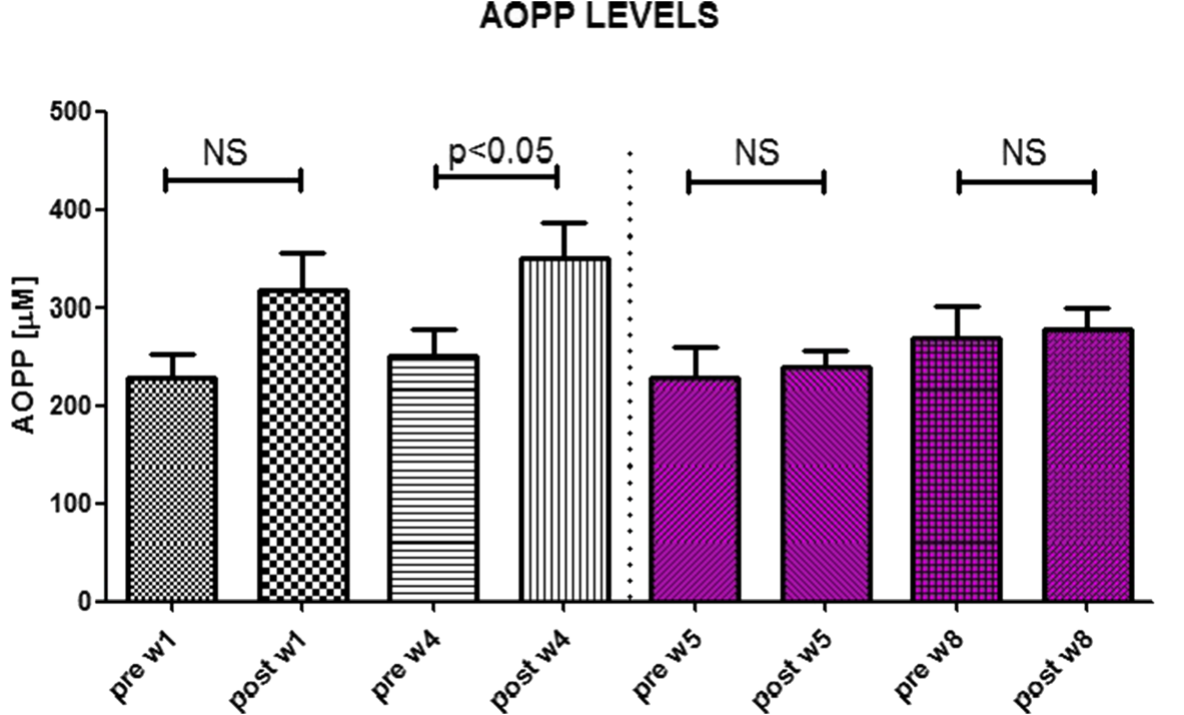
Effects of Carnitine therapy on AOPP levels in patients with CKD treated with intravenous administration of iron. AOPP levels were determined in blood samples drawn 1 and 8 weeks prior and post iron administration (Sodium ferric gluconate, 125 mg/100 ml) During weeks 5–8, these patients received carnitine (20 mg/kg, IV) prior to IVIR

**Fig. 3:**
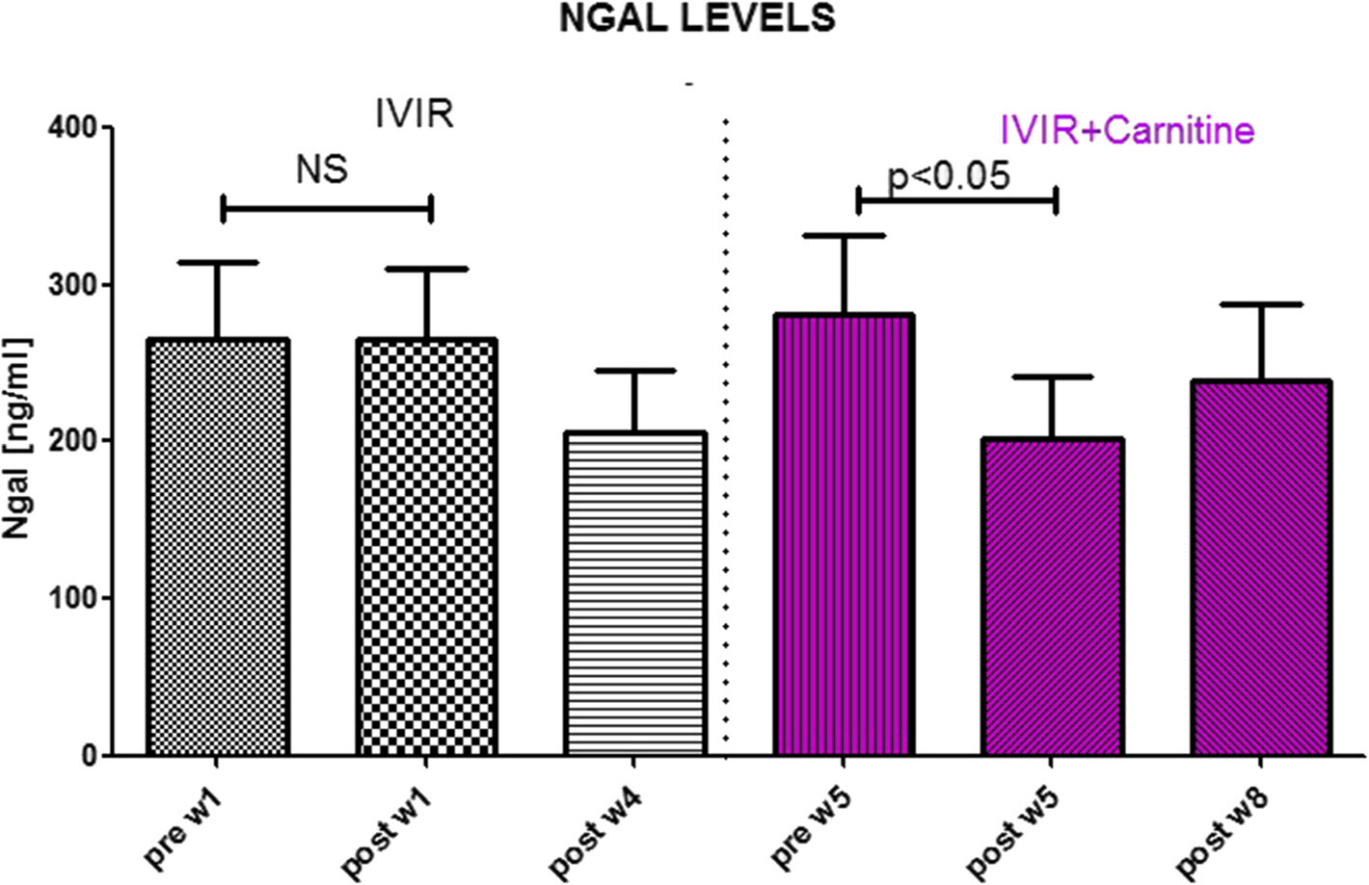
Effects of Carnitine therapy on plasma NGAL levels in patients with CKD treated with intravenous administration of iron. NGAL levels were determined in blood samples drawn 1 and 8 weeks prior and post iron administration (Sodium ferric gluconate, 125 mg/100 ml) During weeks 5–8, these patients received carnitine (20 mg/kg, IV) prior to IVIR

The average age of the studied 26 patients was 63.8 ± 2.84 years (Table 1). Thirteen of them were males and the rest females. Administration of IVIR for 4 weeks did not increase hemoglobin levels (10.23 ± 0.28 vs. 10.31 ± 0.28 g % p = NS) (Table 2), yet worsened the oxidative burden as was evident by elevated plasma AOPP from basal value of 229.1 ± 27.32 to 318.1 ± 39.63 μM (p < 0.05), and 367.8 ± 41.8 (p < 0.05) after 1 and 4 weeks, respectively (Fig. 2). Plasma NGAL levels were not significantly affected by IVIR (265.4 ± 48.55 vs. 265.1 ± 44.7 and 205.7 ± 39.41 ng/ml, after 1 and 4 weeks, respectively) (Fig. 3). Simultaneous administration of carnitine with IVIR resulted in a mild hemoglobin increase (11.18 ± 0.26 g %, p = NS) (Table 2). Interestingly, carnitine therapy abolished the IVIR-induced oxidative stress as evident by preventing the elevations in AOPP (228.7 ± 31.85 vs. 237.4 ± 31.85 and 271.8 ± 23.8 μM after 1 and 4 weeks, respectively, p = NS) (Fig. 2). Furthermore, carnitine therapy decreased NGAL levels from 281.8 ± 49.41 to 201.3 ± 36.63 (p < 0.05), and 239 ± 48.55 ng/ml (p < 0.05) after 1 and 4 weeks, respectively (Fig. 3). No changes in CRP, Serum Cr, BUN, albumin or WBC were observed following IVIR alone or combined with carnitine (Table 2). These findings indicate that IVIR in CKD patients provokes oxidative stress, as evident by elevation of circulating AOPP. Our finding, that co-administration of carnitine with IVIR attenuates the adverse consequences of IVIR, suggests a role for carnitine therapy also in earlier-stage CKD patients.

**Table 2:** Effect of Carnitine therapy on biochemical and hematological characteristics of patients with CKD treated with intravenous administration of iron

Parameter	Pre IVIR-W1	Post IVIR-W1	Post IVIR-W4	Pre IVIR + CAR-W5	Post IVIR + CAR-W5	Post IVIR + CAR-W8
Creatinine (mg %)	2.5 ± 0.31	2.48 ± 0.36	2.38 ± 0.32	2.43 ± 0.31	2.27 ± 0.35	2.59 ± 0.44
Albumin (g %)	4.05 ± 0.08	3.88 ± 0.09	3.83 ± 0.07	4.08 ± 0.07	3.92 ± 0.06	3.98 ± 0.1
BUN (mg %)	42.4 ± 5.4	42.5 ± 6.3	37.3 ± 4.5	43.6 ± 5.7	38.6 ± 5.6	45.3 ± 8.1
CRP	0.74 ± 0.19	0.61 ± 0.13	0.54 ± 0.11	0.66 ± 0.11	0.53 ± 0.09	0.94 ± 0.32
WBC	6.67 ± 0.49	6.65 ± 0.49	5.92 ± 0.43	6.65 ± 0.55	6.25 ± 0.54	6.64 ± 0.58
NEU %	66.7 ± 1.65	67.3 ± 1.66	66.5 ± 1.67	68.9 ± 1.46	66.3 ± 1.85	64.95 ± 1.86
LYM %	22.3 ± 1.3	22.3 ± 1.5	23.2 ± 1.56	20.4 ± 1.26	23.3 ± 1.51	23.65 ± 1.6
Hb (g %)	10.3 ± 0.25	10.0 ± 0.33	10.5 ± 0.21	10.8 ± 0.19	10.4 ± 0.17	11.02 ± 0.22

### Haptoglobin phenotype

**Fig. 4:**
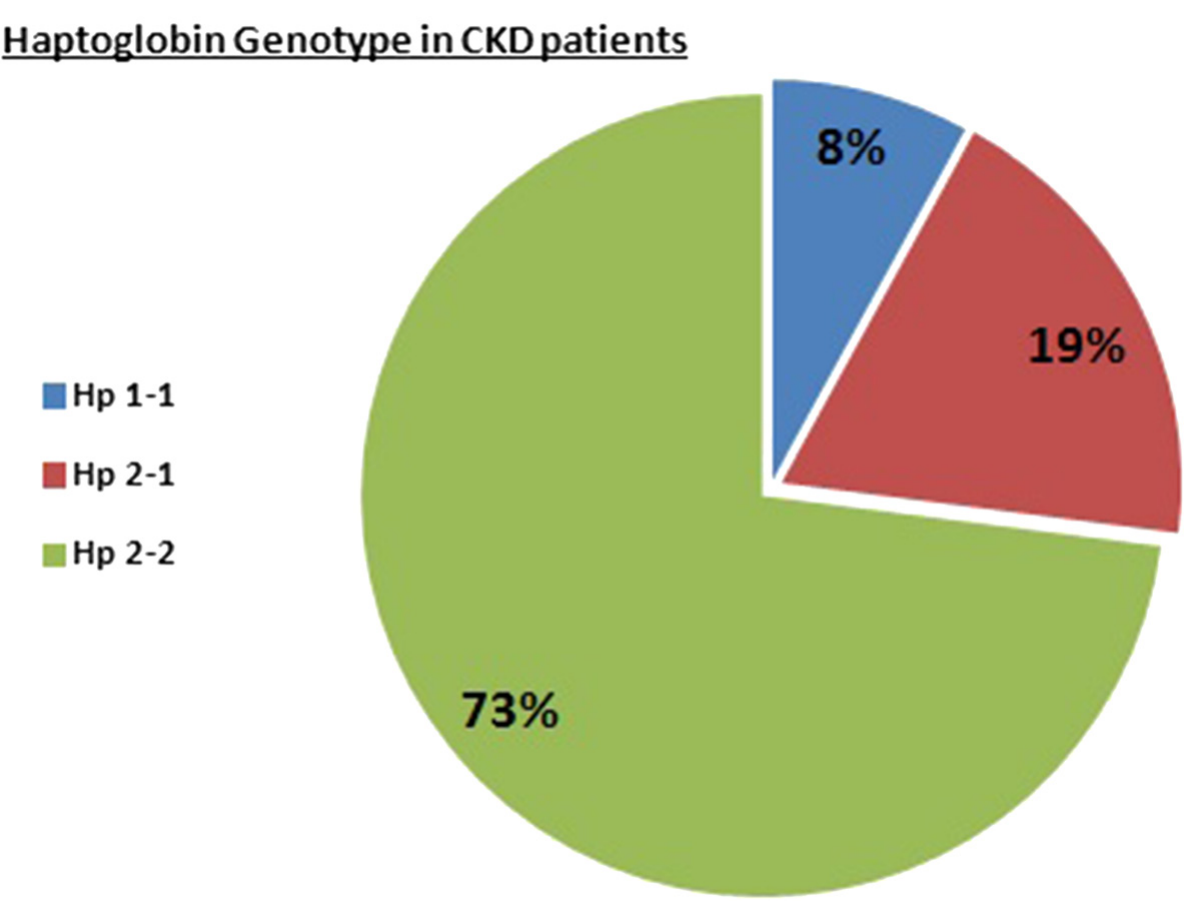
Distribution of haptoglobin phenotype among the studied CKD patients

**Fig. 5:**
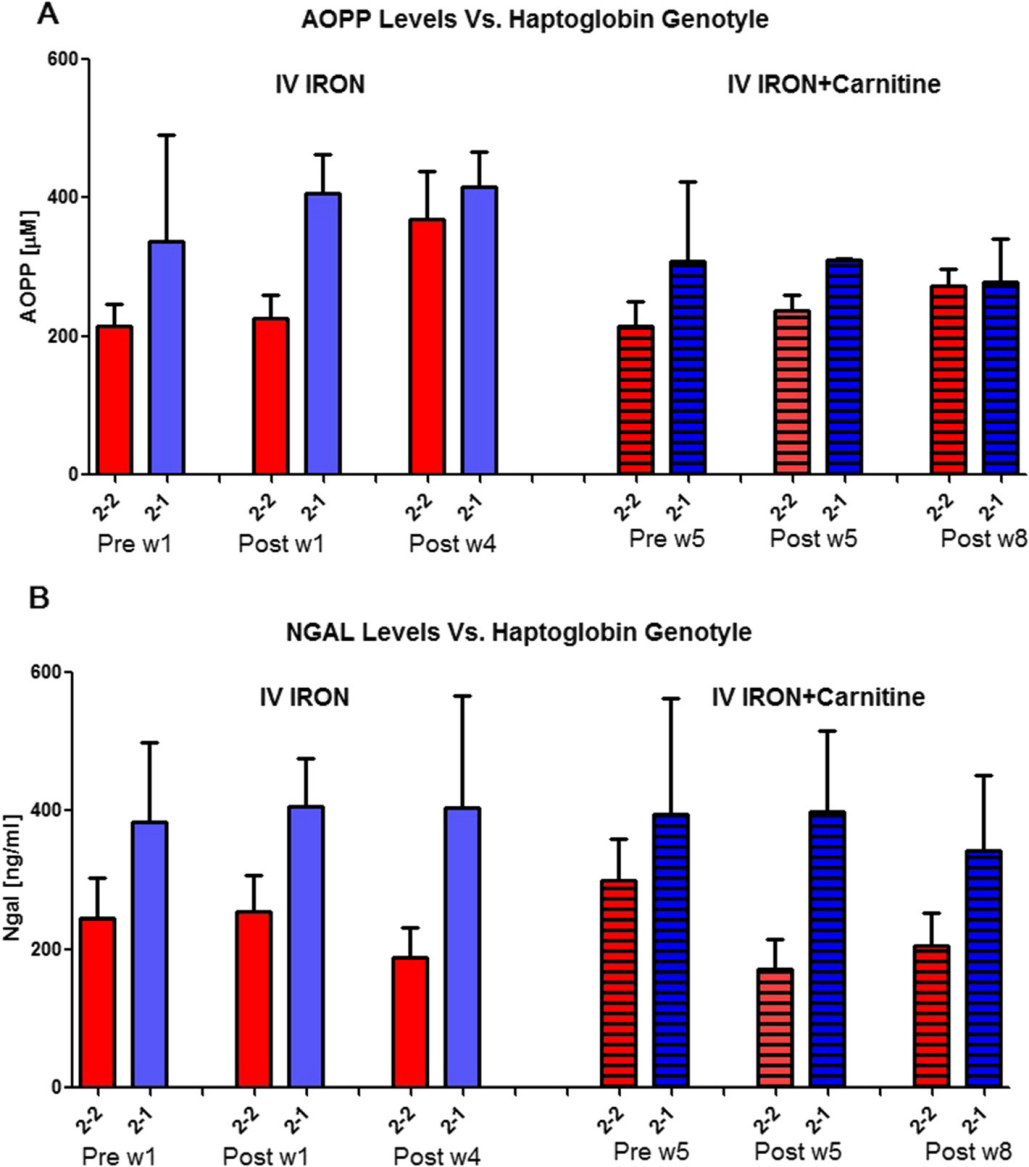
Effect of Haptoglobin phenotype on **a**) AOPP and **b**) NGAL levels in patients with CKD treated with intravenous administration of iron. AOPP and NGAL levels were determined in blood samples drawn 1 and 8 weeks prior and post iron administration (Sodium ferric gluconate, 125 mg/100 ml). During weeks 5–8, these patients received Carnitine (20 mg/kg, IV) prior to IVIR administration

This protocol was designed in order to examine whether Hp phenotype affects iron-induced oxidative stress in CKD patients. Figure 4 depicts the distribution of Hp gene polymorphism among the studied CKD patients. Eight percent of the CKD patients were Hp 1–1, 19 % Hp 2–1, and 73 % Hp 2–2. The alterations in plasma AOPP and NGAL in response to IVIR in the absence or presence of carnitine therapy in these patients according to their Hp phenotype are depicted in Fig. 5. As can be noticed, the increase in AOPP after 4 weeks from IVIR administration was more prominent in Hp 2–2 patients (Fig. 5a). Carnitine therapy reduced plasma levels of NGAL in Hp 2–2 patients administered with IVIR more profoundly than that observed in Hp2-1 subjects (Fig. 5b).

The present study provides new insights into the mechanisms underlying the oxidative stress response to intravenous administration of iron (IVIR) to predialytic CKD patients. We showed that IVIR to these patients provoked oxidative stress as was evident by elevated levels of AOPP. Carnitine therapy significantly reduced the oxidative stress response to IVIR as expressed by lower levels of AOPP. Although, IVIR did not increase circulatory levels of NGAL, administration of carnitine decreased plasma levels of NGAL below basal concentrations. Noteworthy, overwhelming majority of CKD patients are of Hp 2–2 phenotype. The latter were more sensitive to the oxidative stress response to IVIR than others, suggesting that Hp gene polymorphism plays a role in the pathogenesis of IVIR-induced oxidative stress. Unfortunately, IVIR administration for 8 weeks did not increase Hb levels in CKD patients. However, simultaneous administration of Carnitine with IVIR resulted in a mild elevation of hemoglobin concentrations. No changes in CRP, Serum Cr, BUN, albumin or WBC were observed following IVIR alone or combined with Carnitine.

Oxidative stress is a constant feature of patients with CKD and a major risk factor for cardiovascular, neurological and other clinical complications characterizing this disease [4–6]. Intravenous administration of iron in conjunction with EPO is frequently utilized in order to correct the anemia associated with CKD [7, 9, 10]. Although iron administration composes an essential component of CKD therapy, concerns have emerged about its promotion of oxidative stress and related adverse impact. The deleterious pro oxidative effects were extensively studied mainly in dialytic patients [10, 16] and to a lesser extent in pre dialytic subjects [11]. Whether patients with CKD not on dialysis have a similar increase in oxidative stress and, above all, renal injury upon exposure to intravenous iron is unknown. This issue is of critical importance since oxidative stress and renal injury may accelerate renal deterioration and increase the risk of cardiovascular disease [4–6, 44]. Thus, the current study addresses this matter and extends our knowledge in that respect. Our findings clearly show that IVIR administration for few weeks induced oxidative stress also in pre dialytic CKD patients. This was evident by a significant elevation of AOPP, a marker of plasma proteins oxidation. These findings are in agreement with those of Anraku et al. [44] who demonstrated elevated levels of AOPP following IVIR for 4 weeks to dialytic patients. Drüeke et al. [45] demonstrated that advanced oxidation protein products (AOPPs) correlated with iron exposure and carotids artery intima thickness in dialysis patients. In hemodialysis patients, oxidative stress as a result of intravenous iron therapy caused serum albumin oxidation [46]. Similar to our findings, Agarwal et al. [11] demonstrated that 100 mg iron sucrose over 5 min to patients with CKD at stages 3 and 4 increased plasma concentration and urinary excretion rate of MDA, a biomarker of lipid peroxidation, within 15 to 30 min of iron sucrose administration. In contrast to the stimulatory effect of IVIR on AOPP, our findings show that NGAL did not increase following iron administration. NGAL is up-regulated and overexpressed following AKI, and its plasma and urinary levels increased early after renal ischemia in mouse and rat models [34], and following renal ischemic insult in human undergoing pulmonary bypass surgery [32]. The lack of stimulatory effects of IVIR on plasma NGAL in our patients indicates that iron administration did not cause serious AKI [47]. It should be emphasized that plasma NGAL also increases during inflammatory diseases and infections [32], suggesting that the decline in plasma NGAL when carnitine was added could be attributed to the anti-inflammatory effect of carnitine.

So far there is no effective pharmacological therapy for oxidative stress-induced by iron administration. Neither vitamin C nor vitamin E supplementation was found to ameliorate oxidative stress and inflammation in ESRD patients [48]. Likewise, NAC reduced acute generation of systemic oxidative stress but failed to abrogate proteinuria or enzymuria induced by 100 mg iron sucrose to CKD patients [11]. In this context, animal and human data demonstrate beneficial effects of carnitine in experimental models and clinical settings of oxidative stress including hemodialysis [49].

Experimental studies shows beneficial effects of L-propionylcarnitine, a propionyl ester of L-carnitine, in preventing cyclosporine-induced acute nephrotoxicity, reducing lipid peroxidation and significantly lowering blood pressure. L-propionyl carnitine prevented the decline in creatinine clearance (GFR) in cyclosporine chronically treated animals [50]. Clinical studies revealed that ESRD patients treated with carnitine displayed improved physical performance and treatment-related chronic fatigue, cardiovascular disease, cancer, diabetes, and other chronic syndromes, related to impaired carnitine production in kidney disease [49]. In the last decade there are increasing reports describing the beneficial use of carnitine for a better energy metabolism (mitochondrial metabolism). In this context Carnitine increases albumin and protein levels, restores antioxidant defenses, and improves nutritional status, cardiac, vascular smooth muscle, and muscular function [51]. The postulated beneficial effect of carnitine treatment is due to directing lipids towards oxidation and ATP production. Another possible protective effect of carnitine on CKD is its ability to suppress the development of oxidative stress and free radical generation [52]. In line with this notion, our results clearly indicate anti-oxidative properties of carnitine as was evident by reducing AOPP and NGAL levels in CKD patients treated with IVIR.

Finally, our study is the first to our knowledge to investigate the impact of Hp gene polymorphism on the oxidative response to IVIR to CKD patients. Interestingly, most of the studied patients were Hp 2–2 (73 %) whereas only 8 % were Hp 1–1. Similarly, the frequency of Hp2-2 genotype allele was significantly higher in the CKD Taiwanese patients than in controls [53]. After adjustment for covariates, the Hp2-2 genotype (vs. Hp1-1; OR 3.841) remained significantly associated with the development of CKD, together with diabetes (OR 3.131), hypertension (OR 1.748) and dyslipidemia (OR 1.646). Taking into account that Hp plays a role in renal protection, these findings suggest Hp2-2 genotype is an independent risk factor for CKD. Our data support this concept and show that CKD patients of Hp2-2 allele are more prone to develop oxidative stress in response to IVIR than other Hp alleles. Additional larger and longer clinical trials are requested to verify this concept.

## Conclusions

This study demonstrates that intravenous administration of iron to CKD patients provokes oxidative stress as expressed by elevated AOPP levels. Administration of Carnitine to CKD patients reduces the oxidative burden induced by IVIR. The anti-oxidative effects of Carnitine, suggests a role for Carnitine therapy also in earlier-stage CKD patients. Since Hp 2–2 is a significant risk factor for IVIR-induced oxidative stress in CKD patients, the latter may benefit from carnitine therapy more than other Hp genotypes.

## Abbreviations

AOPP: Advanced oxidative protein products
CKD: Chronic kidney disease
CRP: C-reactive protein
Epo: Erythropoietin
HD: Hemodialysis
Hp: Haptoglobin
MDA: Malondialdehyde
IVIR: Intravenous iron administration
NGAL: Neutrophil gelatinase-associated lipocalin
